# Surgical Sketches

**Published:** 1853-01

**Authors:** W. E. Horner

**Affiliations:** Prof. of Anatomy in the University of Pennsylvania, Senior Surgeon at the St. Joseph’s Hospital, &c. &c.


### MEDICAL EXAMINER,

AND

### RECORD OF MEDICAL SCIENCE.

NEW SERIES.—NO. XCVII.—JANUARY, 1853.

### ORIGINAL COMMUNICATIONS.

*Surgical Sketches.* By W. E. Horner, M. D., Prof. of Anatomy in the University of Pennsylvania, Senior Surgeon at the St. Joseph's Hospital, &c. &c.

### *A Military Hospital at Buffalo, New York, in the year* 1814.

(Continued.)

The following general results came within my experience:

1. Buckshot wounds seldom did much harm, vital parts excepted. Those that I saw were principally on the persons of prisoners of war. They approach so near a simple laceration without contusion, that they soon get well. I have now one sticking very tranquilly in an os femoris, as if it had grown there. They are, I understand, peculiar to the American service. They give a more barbarous character to warfare, and probably without an equivalent advantage. This, however, I submit to the decision of military men. The English officers took that view of them, and considered them simply as mangling unnecessarily, but interfering with the effectiveness of the principal ball. Their momentum is too weak to penetrate far; really vital parts are well covered and make but a small portion of the entire area of the body. It would, perhaps, be better for our national credit to discontinue such missiles, at least in war with civilized nations. The only recommendation that I ever heard of them was, that as they inflict a wound, the character of which is of course unknown to its recipient, he retires from the field of battle. They are therefore advantageous simply in multiplying wounds, though the latter may not be serious. If their use be a real advantage in gunnery, they can be thrown by one party as well as by the other, so that things are thus equalized. I have understood that their use at present is not so indiscriminate as formerly, and that they are resorted to principally in close contest.

Should they open articular cavities, then the mischief is of course considerable. Col. Miller of the U. S. Army, in an expedition to Long Point, opposite Fort Erie, had his knee joint perforated in that way, and inflammation and suppuration followed; he finally, after a long treatment, submitted to amputation, but without benefit, as he died shortly after.

2. Bullet wounds which did no injury to the bones, or to the great cavities, generally did well; a slight inflammation and stiffness followed, which went off in a few days. Our most common dressing in these cases, was basilicon, simple cerate, or tallow; poultices or discutients were seldom absolutely indicated. The wound was cleaned by the efforts of nature, in eight, ten or twelve days, it being succeeded by a copious discharge of pus, which gradually diminishing, the wound healed.

3. When the large cylindrical bones were broken, our resort was, for the most part, amputation; we had but few cases, the result of which encouraged us to attempt the saving of others. Out of many cases of fractured os humeri and os femoris which I saw, a majority of the patients died, or were compelled to submit finally to amputation; there being but few instances of perfect recovery from such accidents. The latter circumstance was partly attributable to the largeness of the English musket balls, which almost always produced an extensively comminuted fracture when they struck a hard bone. Amongst the instances of recovery, where the thigh bone was broken, was that of Lieutenant-Colonel M'Neil of the 11th Infantry, and Captain Cilley of the same regiment, and a few privates of other corps.

Col. McNeil was wounded at Bridgewater by a ball which passed through the thigh, somewhat above the patella, and under the tendon of the extensor muscles, breaking in its course some portion of the condyles of the os femoris. The contusion was great. Much inflammation and suppuration followed, but he finally recovered with a joint somewhat stiff. He was a man of vigorous, healthy constitution, and stood more than six feet in height. The probability is that the synovial cavity was not opened; he was attended by another surgeon.

Captain Cilley had his thigh comminuted near the middle by a ball; his attending Surgeon, Dr. Trowbridge,* made four incisions into the thigh, so as to afford room for the discharge of the fragments; they were passed out in a few weeks of inflammation and suppuration, which extended but little beyond the immediate region of injury. A rapid recovery with a useful leg followed, though the length of the latter was reduced three inches.

In the cases of recovery which came under my observation, the thigh bone was broken somewhere in its lower half; this fact corresponds with the testimony of those surgeons who have had the greatest experience in gun shot wounds.† A short time after a gun shot fracture of a large bone, the limb became highly inflamed, and swollen to twice its natural size; sinuses, which opened into the wound, were the almost invariable consequences of this inflammation; after they had existed for a time, the patient's health was destroyed; he became afflicted with diarrhœa and hectic fever, which were very frequently fatal.

4. During the heat of the summer our large amputations were very unsuccessful, so much so, as almost to discourage us from performing them; many of the recent stumps mortified; in others that did not, the patient gradually sank into the arms of death. In the latter, I observed that there was great retraction of the muscles, and frequently the periosteum separated from the bone, for a considerable distance up. Mortification of the extremities from gun-shot wounds, was very common at this period; a variety of tonic and other means were adopted to arrest its progress, and amongst the latter amputation; ‡ nothing succeeded.

*Boston Med. and Surg. Jour. 1838, p. 341.

†See Thomson's Reports of Military Observations in Belgium.

‡See Larrey, (Memoirs of Military Surgery,) who recommends this practice.

5. Wounds attended with much discharge were frequently infested with maggots, the ova of which were deposited by flies (Musca carnaria) in the bed-clothes and dressings. This accident will probably be attributed to negligence, but the most diligent attention from nurses could not prevent it. It was a most serious evil, and frequently involved the life of the patient from the irritation produced.*

It was marvellous to see how deeply these animalcules would work their way into wounds, producing, in some instances, as complete a dissection of the muscles, as if it had been performed by the knife. I learned on this occasion from a common soldier that the expressed juice of the Elder Bark, (Sambucus nigra,) sprinkled on the dressings and bed of the patient, would keep the flies away; and on trial I found it to be so. The suggestion was made in the case of a gallant artilleryman from Virginia, John Horton, in Captain Ritchie's company, and who had suffered the loss of both legs from a cannon shot, followed by amputation. The interest felt in him induced me to place a special nurse to prevent the approach of flies. He was saved from the latter accident, but died in a few days, from his wounds.

6. I saw a few cases of wounds, through the large joints. They were more painful than any other wounds, the patient suffering excruciating torture for several weeks. Somehealed by anchylosis, but we most frequently had in the end to amputate, or to lose the patient, in consequence of sinuses and hectic fever. Sometimes a low delirium took place, particularly in gun-shot wounds of the knee joint, in which the patient died in a few days after the injury. Wounds through the ankle joint were not attended with so much danger.

7. Gun shot wounds of the brain were in some instances very slow in being fatal. John Price, a private of the 1st regiment of Infantry, was wounded at Bridgewater, on the 25th of July 1814; the ball penetrated, transversely, the forehead, passed through the brain above the ventricles, and fractured the opposite part of the cranium; he died on the 2d of September following, five weeks after the injury. Several other examples occurred, in which the substance of the brain was wounded by balls and by tomahawks,

*An analogous fact is quoted from Alanson, by J. Bell.

when the patients continued to live for many days; but I saw no instance of final recovery from such accident.

8. Several persons recovered who were shot through the throat, in such a manner as to endanger the carotid arteries and the important nerves. Amongst them were Brigadier, afterwards Major General Ripley,* and Lieutenant, afterwards Major M'Intosh,† of the Rifle Corps. Both these officers lost the use of their upper extremities for a long time, and suffered exquisite pain in them. The latter discharged the ball, per anum, several months after he had been wounded.

9. Several recoveries from wounds through the thorax took place. Amongst them were Lieutenant Colonel Trimble, afterwards in the United States Senate from Ohio; and an Ensign of the 19th infantry. Bleeding to a very great extent, was used in all these cases. Lieut. Col. Trimble received a ball near the junction of the fifth costal cartilage with the sternum; the ball penetrated the lung and emerged from the back near the angle of the fifth rib. The hemorrhage from the lung by the mouth was most copious, and at each expiration blood poured freely from the external wounds, which of course implied a large collection of it in the pleura. Dyspnœa, feeble pulse, and cold extremities, left him apparently but little chance of life, but on the fifth day the symptoms mitigated. He was bled in the subsequent twenty-two days, six times by his attending surgeon, Dr. Amos Trowbridge,‡ and finally recovered. While a member of the Senate of the United States, he died at Washington in 1822, from a pulmonary affection, brought on by exposure in the preceding season.

10. Wounds of the abdomen, and its contents, were, in several instances, recovered from; amongst such were Lieutenant Cisna, of the 19th infantry, and a Sergeant White. In the first, the ball entered in the anterior part of his belly, below the navel, and

*Case reported in the New York Medico-Chirurgical Journal, by his attending Surgeon, Dr. Allen.

†Case reported in the Eclectic Repertory, by Dr. W. H. Henning, of the Rifle Corps. Lieutenant M'Intosh was at different times under the care of us both. He lived for many years afterwards and was with distinction engaged, I believe, in both the Indian war in Florida, and in the late Mexican War.

‡For details, see Bost. Med. and Surg. Jour. for 1838, p. 344.

came out near the spine; about six weeks afterwards a button was discharged from the wound, near the spine, which had been carried in along with the ball.

Sergeant White's belly was penetrated by a ball, which also took in some fragments off the breech of his musket. The fragments were afterwards discharged at the orifice of the wound, during a free suppuration of it.

11. A case terminated happily where the ball had entered above the symphysis pubis, and came out posteriorly; the fundus of the bladder was penetrated; for several weeks there was a constant dripping of urine from the anterior wound; it at length ceased to flow in this direction, and came in the natural way.

12. In four cases of wounds of the spine, they all had pretty much the same symptoms; being attended with loss of sensibility and motion in all the parts below the injury.

For the first few days the patients were unconscious of their danger, the strength and activity of the upper extremities and parts of the body; and the quickness of intellectual operations, being very encouraging. It was necessary to introduce the catheter twice a day, or more often, in these cases; and to expel the contents of the bladder by pressing above the symphysis pubis. Purgatives were also necessary. In all these cases mortification took place on the sacrum, in consequence of pressure. A great irritability of the stomach occurred in each, a few days before death.

So far as I remember, in those and other similar cases, hiccup has always attended, and also delirium, before the final scene.

13. In amputations of the leg, to get the flap from the integuments, on its posterior part, was decidedly advantageous, in some respects, regarding the healing of the stump. If, instead of this, a circular flap was made, the part of it covering the sharp edge of the tibia was most generally ulcerated through, and the ulcer remained exceedingly painful and absolutely incurable, till the absorbents rounded off the tibia. This disadvantage was very striking with the circular flap adjusted transversely instead of vertically, *i. e.* antero-posteriorly. The flap extending from behind is, however, apt to separate, or rather not to attach itself to the muscles; and, in such case, when suppuration came on, it retained the matter, and sometimes ulcerated through. My subsequent experience has led me now to prefer the circular cut with the vertical adjustment, and a bevel cut upon the spine of the tibia saves the absorbents a long work in rounding it off.

I saw an attempt to make a flap of the gastrocnemius and solæus muscles, on the principle recommended by Mr. Hey and others. It succeeded badly, the flap became very much inflamed and swollen, left entirely the surface of the stump, and could not be brought back; it gave the limb the appearance of a club. After a variety of means was fruitlessly tried to bring about cicatrization, a second amputation became necessary in order to save the patient's life.

A similar attempt on my own part since, in the Philadelphia Alms-House hospital, though not attended with such bad consequences, was very unsatisfactory, from the difficulty in making the flap unite.

Two amputations of the inferior extremities, a thigh and a leg, were performed by an inexpert surgeon, in which no flap was left; the patients recovered, however, after a very long confinement. The stumps became conical from the retraction of the muscles, and a second sawing of the bones was requisite to the cure.

Two amputations at a delayed period, that is when inflammation had set in, were performed. The suffering of the patients was much beyond the ordinary agony. The quickly fatal results proved the necessity of attending well to the rule, that such operations are never to be performed for severe projectile wounds, except in a few hours after their reception; or after several weeks have elapsed, and the symptons of inflammation are entirely gone.

Soldiers are much disposed to repress the expression of pain, considering it unmanly. When this natural mode of easing the circulation of the lungs by groans and expiration was withheld, which can only be done by holding the breath, I came to the conclusion that it was highly disadvantageous to the individual. Chewing a bullet is a resort which should also be discountenanced. The introduction of ether since that period, will now make these cautions less necessary. The amount of pain excited in different persons varies very much. I assisted at an amputation of the leg of a soldier who was smoking tranquilly during the whole operation, his ease not seeming to be an affectation.

I did not see a case of recovery where amputation had been performed on both legs, or on both thighs, in one subject; but I saw a few recoveries *where* a leg and an arm, or two arms had been resected. Several double accidents of this kind happened during the seige of Fort Erie. In one instance, a cannon ball entered a hospital, and ranged along the feet of a row of bedsteads, carrying away several legs in its course. In another instance, a young rifleman, of eighteen, belonging to Captain Irvine's company, had both arms taken off above the elbow joint, by a cannon shot. The ball being nearly spent, he then started in pursuit of it, not appalled by what had happened, but continuing to kick it as an enemy until its motion gradually subsided. His gallantry and coolness on this occasion, procured him the notice of several distinguished officers. I saw one case of recovery from amputation at the shoulder joint. This operation was performed by Dr. Gales, of the 23d infantry, whose services and intrepidity gained a high distinction.

14. Some cures, from extensively lacerated wounds, took place; amongst them, J. King, a private in Captain Biddle's company of artillery. He had the whole posterior part of his thigh, from the buttock to the ham, torn up by the fragment of a shell, and the muscles laid bare, as in a dissection. This extensive wound, in which there was a great loss of integuments, cicatrized, and got nearly well in three or four months. A private in the Infantry already alluded to, was scalped in a circular line, just above the tips of his ears. Granulations arose from the exposed surface of bone and pericranium, in a few days, and cicatrized very kindly; the discharge of pus was, for a long time, very considerable.

15. In one case I succeeded in a very rapid cure of fistula, in the lower extremity, produced by fracture in the lower part of the tibia. An intense and extensive inflammation of the whole limb followed the injury; this was succeeded by the deposition of matter all up the leg, and extending even above the knee. When the suppuration was pretty well established, by pressing carefully the matter from the sinuses for two or three days, so as to empty them completely, I succeeded in obtaining a radical cure, by placing compresses on the course of the sinuses, and securing them by a tight roller applied from the foot to the groin. The fracture did very well.

This manner of treating inflammation of the extremities, has since been very fully elucidated by the celebrated French Surgeon, Velpeau, in his writings upon the principle of what he calls systematic compresses.

16. I treated two cases of traumatic tetanus; the one was produced by a gun-shot wound through the pectoralis major muscle; the other by the same kind of wound through the sole of the foot. The first was managed with large quantities of laudanum; the patient died. The second was treated with laudanum also; and according to the plan proposed and adopted by Larrey, the surface of the wound was cauterized with a red-hot iron. A temporary remission was produced in the spasm, but it returned, and the patient died the day after the application.

Such were the general observations resulting from the surgical occurrences which came under my personal notice.

As in most active campaigns, the duties of the surgeon were too urgent to allow time for the taking of very complete notes on cases. Hence, general recollections have to be substituted, to a large extent, instead of precise tabular statements. At this period of time, when the actors of that day have so generally passed off the stage, it is a matter of sincere regret to me that so little has been published by others; and from recent enquiries at the War Department, it appears that no reports are there to supply this deplorable defect. It is to be hoped that the few medical survivors of this remarkable campaign may yet be stimulated to give in their full experience by publications, or at any rate make a deposit of it in manuscript at the Medical Bureau of the Army. How precious would such details of the American Revolution be; yet there are few or none.

The following account of the diseases and weather will probably be read with interest:

* *Report of Hospital Surgeon* LOVELL, *of the state of diseases among the Troops on the Niagara Frontier, during the Campaign of* 1814.

"The troops engaged in this brilliant campaign on the Niagara, began to collect there about the beginning of April, under the command of General Scott. They were encamped on an eminence north of Buffalo

* From Medical Sketches of the Campaigns of 1812, '13, 14, by Jas. Mann, M. D., Hospital Surgeon, &c. Pp. 160 et seq. Dedham, 1816.

village, having a thick wood in front, which extended to the bank of the river, the ground being in part swampy and wet. On the left of the encampment was a large marsh, extending from the high ground to the margin of the lake. The winds from the lake, at this season, were remarkably cold and chilling; resembling, in sensation, exactly the east winds which prevail on the Atlantic during the spring; and had an astonishing effect upon vegetation. The trees around the encampment having the appearance of winter, while those five or six miles from the lake shore, were covered with verdure. Notwithstanding this, the troops were remarkably healthy; only one or two deaths occurring before they crossed the Niagara, on the 3d of July—even the demon diarrhœa appeared to have been exorcised by the mystical power of strict discipline and rigid police.

In June a number of new recruits joined the army; and several were collected from the various hospitals; the latter principally composed of the miserable refuse of society, who never had energy enough to demonstrate that they lived, and scarcely enough to prove that they existed. With these last detachments, arrived our old acquaintances, which, however, were easily checked; and much seldomer returned, than in any former campaign. This was undoubtedly to be attributed to the improvement in police.

During June, the weather became very warm, and a thick fog arose from the marsh and woods at sunset, and remained for some time after sunrise. During this month, intermittent fever, acute rheumatism, and typhus fever were the prevailing complaints. The intermittents were very irregular and obstinate. Arsenic, which was the sovereign remedy the last year, on this frontier, had now very little effect; while the bark, which then failed, was now generally successful. Some obstinate cases, in which every thing else had failed, were cured by the sulphate of copper. Three patients, who had tried most of the remedies with which we were supplied, without effect, cured themselves at once, by taking a pint of brandy undiluted, in which was mixed a large quantity of ground black pepper, on the accession of the cold stage. This was not followed by inebriation nor any appearance of undue excitement. It led me to use opium in much larger quantities than I had been accustomed. It was begun with four or five grains at a dose, and increased until some stimulating effects were produced, or the disease cured. The success of this prescription was very great during the whole season. In fine, of the remedies used this season, emetics had but little effect, even at first; and the mineral solution scarcely any—bark succeeded in the majority of cases; and opium very seldom failed. A few obstinate cases were checked for several periods, by the application of tourniquets to one leg and one arm; the disease however recurred; the tourniquets then had no effect; but remedies, which had before failed, now succeeded, after the interruption thus produced in the morbid associations.

Rheumatism, during the whole war, generally put on a remitting form; this was particularly obvious whenever intermittent fever prevailed, and more especially this season. Bleeding was but seldom necessary; after a brisk cathartic, bark was given in the quantity of from four to eight drachms during the remission, and a large dose of opium on the accession of the fever; and always in sufficient quantity to relieve the pain. This treatment was very generally successful. I was induced to try it, in many cases, where the remissions were very slight, and generally effected a cure. In these, however, bleeding or purging were premised, which produced more perfect remissions. In short, I considered the bark and opium the remedies for rheumatism, particularly when intermittents prevailed, and for the most part succeeded.

Many of the cases of typhus, about the end of May, were remarkably severe. The most prominent symptoms were great prostration of strength, and delirium; of the species not attended with symptoms of great arterial action in the head, local applications as usual having no effect upon it. Symptoms of recovery were not observed in these cases, until the end of the third week. The treatment adopted was strictly that of Fordyce, and recovery took place in every instance.

On the first of August, a general hospital was established at Williamsville, eleven miles east from Buffalo. The number of sick, during the remainder of the season, at this place, varied from 3 to 400; the number of wounded being somewhat greater.

The troops suffered much during the seige of Fort Erie; and soon after it was raised, the rainy season commenced. Dysentery and diarrhœa were the principal diseases. I became fully convinced, after a fair trial of every medicine to be obtained at this place, of the decided advantage of ipecacuanha in various forms and doses, to any other remedy. The remarkable effects of this medicine, which Fordyce considers as acting specifically in typhus fever, led to the conclusion, that the febrile symptoms attending the latter stages of diarrhœa were in fact a true typhus, supervening upon the former complaint. Hasty in his treatise on dysentery, he speaks of several complaints, which are often combined with typhus fever; and are then generally contagious; and I had observed that the nurses of the wards, where diarrhœa prevailed, were often attacked with typhus, accompanied with diarrhœa, or a great tendency to it. Decided benefit had often been observed from small doses of ipecacuanha, with mucilaginous drinks, in an irritable state of the stomach and bowels, which appeared to be owing to a degree of inflammation extending through the mucous coats; and not attended with febrile symptoms; and it is probable that the good effects of the remedy, in the cases now referred to, were in some measure to be attributed to this mode of operation. Intermittent fevers and rheumatism prevailed during the whole season, and varied but little from the cases in May and June. The cases of typhus among the regular troops were generally mild.

About the end of September, a large detachment of militia crossed the Niagara, under General P. B. Porter. Diarrhœa, typhus and idiopathic dysentery very soon made their appearance among them; the two latter were extremely severe. As these patients were not sent to the general hospital, until they had been sick for some time, I saw only the latter stages of these complaints. The dysentery was at this period very obstinate; the bloody discharges and tenesmus incessant, and the prostration of strength as usual most dangerous. In this state, relief was very generally obtained from injections of a decoction of ipecacuanha, sometimes combined with laudanum; at others, the irritability was first reduced by an injection of laudanum alone. The decoction was often rejected immediately; it had, however, some effect even then, so that by repeating it several times, it would finally remain, and give relief. Blisters to the abdomen often had a very good effect; but no application to the part appeared generally to prove so beneficial, as a poultice of slippery-elm bark to the whole abdomen, often repeated. It relieved the tenesmus, and produced a gentle diaphoresis, which was promoted by warm mucilaginous drinks, a mixture of tinct. opii. and tinct. ipecac. This was the only treatment found beneficial in the latter stages of this complaint, and it very generally succeeded. Typhus, among the militia, was very severe. Patients were seldom sent to the general hospital, until the third week of the fever; and the treatment had been different, as the whims of the attending surgeons. The most usual practice, however, among them, was to blister the patient almost from the crown of his head to the soles of his feet; so that the chief difficulty was to remove the irritative fever induced by this *empirical, slovenly* practice. In some, calomel had been employed, but generally without any obvious effect, except increasing the danger of the patient. At this stage of the complaint, and under these circumstances, no general method of treatment could be adopted, except remedying the mischief which had been done. The cure was principally attempted by removing every cause of irritation, as appeared most urgent, and trusting to nursing and nourishment. Under this plan many appeared to be in a fair way of recovery; but in the course of the fourth week, a small circumscribed spot of inflammation showed itself in the face, generally, near the angle of the mouth. In a few days the whole side of the face swelled; this tumor was hard and pale, resembling the color of a white swelling of the joints. It was not in the seat of the parotid gland, but anterior to the branch of the lower jaw, and was attended with a most profuse and fetid salivation, apparently from irritation communicated along the salivary duct, as the liver and gall-bladder are excited by the chyme. In a few days more the red spot began to assume a livid appearance, and symptoms of incipient mortification. In a short time, the mouth was literally extended from ear to ear, exposing the backmost grinders on both sides. All the remedies usually employed in this species of disease, were employed without visible benefit. The only article which appeared to produce any good effect was charcoal, which, however, seemed only to prolong the sufferings of the patients. Three attacked with this affection had severally so far recovered as to have a good appetite, and sit up a great part of the day. Their strength and appetite held out surprisingly, after mortification had taken place. I have since seen two instances among citizens; one in Boston, on a young boy. He had so far recovered as to sit up; he took nourishment with a good appetite, and every symptom of fever had disappeared; when, about the middle of the fourth week, the swelling, salivation and mortification took place, and shortly he sunk. It should be added, that in the majority of these cases not a particle of mercury had been used in any form."*

*The same disease was observed by Surgeon Purcell in the Military Hospital, Burlington, Vt., in the autumn of 1814. See Mann, loc. cit., p. 164.

Several interesting cases of wounds occurred in our hospital after the battle of Chippewa; many operations were performed by the regimental surgeons on the field of battle, but a majority of them were left for the hospital staff. I shall relate a few cases, with the success attending their treatment.

CASE 1.—A private in the 11th regiment of infantry, received a wound from a grape shot in the right breast, between the nipple and sternum; the ball passed under the sternum, obliquely downwards, and emerged about four inches from the sternum on the left side, beneath the edge of the pectoralis major. The first time I saw this patient was on the third day after the reception of the injury; he said he had bled a great deal, and his clothes still showed the marks of considerable hemorrhage. I presumed that the internal mammary artery was cut or ruptured; however, as the wound had ceased to bleed before I saw him, the condition of the artery did not excite much attention.

The patient's pulse was feeble and quick, and he complained of great stiffness in his breast; his breathing was regular but hurried; I prescribed a light diet for him, and applied the common adhesive plaster to his wound on each side. I was much struck with the circumstances of this case, for the ball had passed between the sternum and mediastinum without producing a solution of continuity in either, which, had it injured the latter, would have been known by the state of respiration. I went to visit him on the fourth day, having obtained the advice and assistance of Dr. Thomas, hospital surgeon. The patient's pulse had become full, he had a slight cough, and complained of wandering pains in his breast, superadded to those occasioned by the wound. The depleting plan was here evidently indicated. I bled him to the amount of sixteen ounces, and dressed the wound as usual; in the afternoon I bled him again, as the symptoms had not been mitigated. By bleeding four or five times more, once a day, or on alternate days, the inflammatory symptoms were thoroughly subdued, and he no longer complained of his breast, except in the wounded part.

About the tenth or twelfth day the suppuration was so profuse as to require three or four dressings in the twenty-four hours.

His appetite finally returned, and his excretions were regularly performed; he was, however, from being a robust man, much reduced. As the suppuration diminished, the granulations sprung up; in about fifty days the cicatrix was completed, and his health, in a considerable degree, restored.

Case 2.—Sergeant Smith, of the 11th infantry, was wounded the 5th of July, in the breast, by a musket ball. The ball entered on the left side, between the sixth and seventh ribs, and came out near the spine on the same side. I saw him on the third day after the injury. He then labored under oppression of breathing, and his muscular strength was much prostrated. The sides of the wound were tumefied and inflamed; I, therefore, could not make a satisfactory examination of the direction of the ball. I supposed, however, from his anxious and hurried breathing, that the lungs were wounded. I bled him to the amount of a pint, and dressed his wounds with yellow basilicon. On the fourth day I bled him again; this bleeding seemed to relieve him much. I enjoined on him the strictest antiphlogistic diet that the nature of our hospital stores admitted of.

By some new arrangement in the wards of the hospital, he was left out of my list, and transferred to my assistant, Dr. Coltrin; I therefore did not see him again for eight or ten days. At that time the wounds in his side were discharging matter very profusely, and a few granulations had risen up.

Air was inhaled and exhaled through the wound in the fore part of his breast, but not through the posterior wound. He believed himself to have been struck by two balls at the same time; if this be the fact, it will account sufficiently for the air rushing only through the anterior wound. About the last of August I saw him again; he was then emaciated almost to a skeleton, and had a harassing cough, with purulent expectoration, which threatened to terminate his existence in a few days. He continued in this unpromising way for several weeks longer; the wound in the fore part of the chest healed up, but that in the back continued running. His wounds, during this period, had been dressed with yellow basilicon or cerate, and his cough palliated by demulcents. The cough left him about the first of November, but his extreme emaciation and weakness continued

From this time he began to recruit a little, and in fair weather sometimes ventured out of his apartment. We now entertained hopes of his recovery. About the first of December, the wound in his back discharged but little, and was nearly healed. He was suddenly seized with lancinating pains near the wound, and spasms of the intercostal muscles and diaphragm, which sometimes suspended his respiration for a minute or more. Our hopes now vanished, and the attendants were frequently in the act of laying him out for dead.

Dr. Coltrin and myself visited him in concert. On examining the wound in his back, I perceived an obscure fluctuation near t. Having made an incision with my lancet, a few spoonfuls of healthy pus were discharged, and relief obtained for the time. He was ordered to take twenty drops of tinct. thebaic. at night. The next day, a small spiculum of bone was taken out. The spasm and pain now left him, and he recruited again. The wound in his back healed in about ten days, and he had a second return of this affection, as alarming as the first; a similar treatment was pursued, which relieved him. On the 24th of December, he was sent to the general hospital, at Williamsville, in a convalescent state, having had no subsequent return of his paroxysm, and bidding fair to recover entirely.

Case 3.—A private, in the 9th infantry, had his radius broken about the middle, by a musket ball, which passed obliquely through the arm. He was admitted into the general hospital on the third day after the accident. The arm was then slightly tumefied and painful; only a common dressing, with splints, was applied to it. Between this time and the twenty-fifth day, frequent hemorrhages had occurred, which were commonly suppressed by a compress of square pieces of muslin, confined with a roller. The arm had become greatly tumefied, probably to three times its natural size, and a copious suppuration had followed, which relieved the inflammation and pain, but the patient's health was much impaired by diarrhœa and hectic symptoms. Opium and bark having been tried in vain, to arrest the latter, it was determined, on the thirty-fifth day after the accident, to take off his arm. I amputated it just below the insertion of the deltoid muscle. On the fourth day afterwards, I undressed it, and was much chagrined at finding that the adhesions of the flap to the muscles had been prevented by a number of maggots finding their way through the dressing into the stump, notwithstanding every precaution had been taken to prevent such an occurrence. I cleared them out by injecting warm water and spirits of turpentine. On the fifth day, a new brood had come in, and insinuated themselves into the interstices of the muscles, producing considerable pain and irritation. The muscles had retracted, and left the bone jutting out about an inch, completely denuded of its periosteum. A very slight suppuration occurred. The patient's health became worse; for, besides an aggravation of the aforementioned symptoms, a comatose condition was present. I sawed off the protuberant extremity of the bone, cleaned the wound with warm water, and ordered the tonic and stimulating treatment to be strictly pursued. Nothing seemed to relieve him, and on the tenth day after the operation, and the forty-fifth from the reception of the injury, he died.*

CASE 4.—John McGulrick, aged 45, of a dark complexion, and full habit, a private in 100th British regiment, 4th company, was wounded and taken prisoner at Chippewa, on the 5th of July, 1814. On the 7th of the same month, he was brought to the general hospital at Buffalo, with the rest of the wounded of the two armies. On examination by the superintending surgeon, the thigh was discovered to be fractured by a musket ball, about six inches above the knee joint. The limb was much swelled, and extremely painful from the bandage applied in the first dressing being stiffened with blood, and too closely rolled. He was ordered to live on light food, have the limb frequently moistened with lead water, and secured with a bandage and splints. A dressing of cerat. simp. was used.

In consequence of the great number of wounded, the surgeons and mates of the general hospital were unable to attend to all the patients, and it became necessary to employ some of the neighboring practitioners. It fell to the lot of this man to be placed under the hands of one of them, and I did not see him again till about the middle of October; I am, therefore, unable

* This case is inserted in order to show the effects of local irritation on a stump.

to say what treatment was pursued in the interim ; from circumstances, however, I am induced to believe it was not of the most judicious kind. About this period, the services of the aforesaid practitioner being no longer wanted, he was discharged, and the soldier placed under the care of my assistant, Dr. Coltrin. On visiting the patient, he was struck with the peculiarity and precariousness of his situation, and was sensible that the preceding surgeon had not done his duty. The case presented such difficulty that he requested assistance.

Oct. 16th. We visited the patient in concert, and found the following symptoms: He was reduced almost to a skeleton, there being little else but skin and cellular substance on his bones; his pulse quick and feeble; a wasting diarrhœa had been on him for several weeks; he was subject to profuse sweats at night, and had no appetite. To these general symptoms was added a most unfortunate condition of the broken limb. The thigh was shortened about four inches, in consequence of the extremities of the broken bone being allowed to pass each other; the orifices, where the ball had entered and made its exit, were still open, and discharged daily a large quantity of thin semi-transparent pus; a fistula was found running nearly up to the trochanter minor, and a large ulcer had formed on the sacrum from continued lying in one position.

Weighing all these circumstances, we were not slow in pronouncing an approaching dissolution: had our opinion been otherwise, we should have determined immediately on amputation. There seemed so little chance for the patient's life, that nothing else could be done but to pursue vigorously the tonic plan; this we did, more from a sense of duty than from hopes of success. He was ordered to take Peruv. bark four or five times a day, and also a pint of port wine.

℞. Kino. gr. xv. To be taken three times a-day.

It was too late to think of restoring the limb by extension and counter-extension, as an adhesion had taken place between the bones, and an artificial joint had formed. We, therefore, only directed the limb to be dressed with basilicon twice a-day, the matter to be carefully pressed out of the sinus, and a spiral roller, with a compress on the fistula, to be applied from the foot to the groin. The ulcer on the back was dressed also with basilicon.

This treatment was persevered in, with the addition of bitters, till the 1st of November, and with no amendment on the part of the patient; we, however, were pleased, and thought him fortunate in not dying.

The patient adhering to life with such tenacity, made us agitate the propriety of amputation, and we almost made up our minds for it, but as he was in the same precarious situation, it was thought advisable to wait a few days longer, as they would probably determine his fate, or produce an amendment.

Nov. 5th. The same treatment continued to this date; the patient no better, diarrhœa and hectic symptoms rather more urgent. As a dernier resort, we this day determined on amputation; everything was prepared, and the knife placed in the hands of Dr. ——, hospital surgeon, who, not belonging to the hospital, as a mark of attention, had been invited to perform the operation. He was dissuaded from it by Dr. ——, of the —— infantry, also a visitor, who, forming his opinion from the present condition of the patient, without taking into consideration the preceding circumstances, denounced the operation as fatal to the patient, and involving the reputation of the surgeon; advising us, at the same time, to pursue the tonic treatment. But a full trial of that had been already made. I was myself inclined to believe, that the operation might probably be fatal immediately, or in a few hours afterwards: but as I considered it the only possible means of relief, and had maturely reflected on the advantages and disadvantages likely to result, it was with reluctance that I submitted to the opinions of the consulting surgeon for the present.

Dr. Coltrin and myself having both acquired confidence from seeing the happiest issue from amputations, under circumstances similar, but not so aggravated, determined, let the event be what it might, on performing the operation by ourselves, without consulting or letting it be known to any other surgeons; we accordingly proceeded to the operation on the

10th Nov. At this time the patient's strength was completely prostrated, the diarrhœa and hectic fever still continuing in all their force; the sores on the back and thighs were no better. The limb was amputated about two inches below the trochanter minor; the patient lost but little arterial blood, and bore the operation beyond our expectations. He was put to bed and ordered T. Opii, gtt. xl.

Nov. 11th. The patient somewhat better, rested tolerably well last night, less troubled with the diarrhœa.

℞. Pulv. Cort. Peruv. coch. min., still to be taken four times a day, and Elix. Vitriol, gtt. xv., to be added to each dose. A pint of port wine a day continued. Allowed to eat any thing he fancied, which could be procured.

Nov. 12th. Rather better, medicine continued.

Nov. 13th. The symptoms considerably ameliorated, appetite excited, had but one stool yesterday, and one last night, nocturnal sweats less profuse, rested well, medicine continued.

Nov. 14th. Mending perceptibly; the first dressing was removed from the stump; it had partially united by the first intention, and was beginning to suppurate finely. The ulcer on the sacrum throwing out healthy granulations, and diminishing in size.

Nov. 20th. The patient much in the same state as on the 14th, except that the powers of life were so feeble in the sound limb, that a dry gangrene came on the big toe, and the two adjacent toes, being probably induced by the cold state of the atmosphere.

The extremity was cold and without perceptible pulsation, from the toes to the knee. The nurse was ordered to wash it twice a day with hot brandy, and apply a flannel roller from the toes to the groin; medicine continued.

Dec. 1st. The patient's health and strength considerably improved, appetite good. The dry gangrene still continued on the toes, without any effort of nature to separate the mortified parts; the heat of the limb was restored after a few applications of the hot brandy; pulsation perceptible in the anterior tibial artery. The stump and ulcer doing well, the latter nearly healed. We now considered the patient almost out of danger. Prescription of the 11th November continued.

Dec. 20th. The patient completely exempt from apparent danger, and convalescing rapidly. He had picked up a little flesh, his appetite good, diarrhœa and hectic symptoms entirely gone; but one or two stools in the twenty-four hours; the ulcer on the sacrum healed, and the stump nearly so. Bark, Elix. Vitr. and wine continued.

Dec. 23d. The general hospital being broken up at Buffalo, this patient was sent, with others, to Williamsville, eleven miles off; no question of his recovery was then entertained, and the only unfavourable circumstance was the gangrene on his toes, which still continued, nature showing an indisposition to separate the living from the dead parts. He arrived at his journey's end without accident. The final conclusion of this case I have to be informed of, as I left the frontier the following day.*

Gun-shot wounds having got their name originally from a superstition connected with them, that is of their being poisoned or placed under some peculiar influence from the material projecting the ball, they have been considered as forming a class to themselves, and a special treatment was introduced, now to a great extent abandoned. If the term itself were laid aside, and we had a class of wounds called Projectile, surgical phraseology would be improved by accommodating it to the sentiments now generally entertained. The mere instrument of projection in its own nature has no influence, further than its special force, upon the body projected. At the present day, when steam is so much employed in navigation and in machinery; from the frequency of accidents from its explosion, we might, upon the principle of the nomenclature adopted, have a class of steam-shot wounds, and formerly there could have been one of balister-shot wounds. It is probably the more requisite to have a reformed nomenclature in the present day, because really great modifications have been introduced into the manner of inflicting wounds. The injury from musket balls formerly occupied almost exclusively the mind. Now artillery is so much used in combat, that its grape and its cannister shot figure largely. The projection of hollow balls calculated to explode at certain points, and by their fragments produce destruction, has also advanced much in use. And lastly, the modes of injury connected with the industrial pursuits of society, (as the disrupture of

* The amputated part of the os femoris is now in the Wistar Museum, and presents a striking example of the effort at union by callus thrown out laterally.

machinery, and the tearing and crushing of railroad cars,) stand much in the category of projectile wounds, though not exactly according to our pre-conceived idea of them. The whole of the preceding category belong in their phenomena to wounds resulting from laceration and contusion blended together. The entire train may indeed be said to commence with the buckshot used in the American service, and to end in the explosion of the radiating fragments of a bomb shell, or in the most extreme form of railroad accidents. The injury from the first is very insignificant, unless it touch a vital part, as the brain or a large vessel; while the injury from the two last, though falling upon parts easily spared from the system, yet come with such destructive violence, that the system is unable to rally from it, and hence death is most lamentably frequent, without our being able to point out any very intelligible cause. We call it, however, a *shock* to the system.

If it were in preceding times difficult to give a formula, which represented universally the progress of wounds from projectiles, (gun-shot,) and the exact mode of treatment, that difficulty it is seen by the above is much augmented, at the present day. The enquiry has therefore to be made conformably, and resolves itself as follows:

The circumstances of force and resistance, being the same in both instances, what are the phenomena attending the entry and exit, or lodgement of spherical shot or bodies, connected with the material forming the latter? What of angular bodies, made so by design or by explosion?

How is their passage modified by the various degrees and angles of resistance?

What are the most appropriate dressings in each case?

What are the different constitutional effects?

What are requisitions for amputation, and at what time after the injury should it be performed? This is perhaps the most important and pressing question of modern surgery; from the revolution, I say, which has taken place in the projectiles of modern warfare, and in the use of transporting and of manufacturing steam machinery. Its paramount interest is seen every day. Formerly a stump, either in arm or in leg, was one of the rarest of sights in our streets, and was sure to obtain attention from its infrequency; now it is so common that we do not think of it in passing. A death after an amputation was formerly scarcely looked for except from the general condition of the patient at the time; now it is one of frequent occurrence. The question, therefore, has been most forcibly elicited, what are we to do with limbs extensively crushed and lacerated? Are they to be cut off before re-action—are they to be cut off upon re-action—are they to be left as we find them, and to await the sanative process of nature, which will cause to slough off a part whose life can no longer be retained? Or are we, in case of restoration being hopeless, to make a simple muscular resection of the part, at the poin which is the most disjointed from the rest, and to leave the resection of the bone and its squaring off, for a future time? It is impossible for any one man to find in his own experience a solution for al these questions, but a collective attention of the profession from all quarters of the world, in making a simple report of each case, would enable us to see whether the existing rule of speedy amputation is to be observed; or whether a new state of things, by its greater frequency of occurrence, may not invite to another rule of practice, by affording a higher and a more discriminating experience.

In hopes of eliciting from other quarters their own experience, and by that means obtaining a total revision of the ground of surgery in its relation to wounds inflicted by projectiles, I shall now proceed to present my own observations and conclusions.

In regard to the lighter conditions of projectile wounds, the following phenomena and proceedings may be stated:

When a wound is first inflicted, say by a musket ball, because it is the most frequent cause of injury, a round hole is made, into which the surgeon can easily introduce his finger: in twelve or twenty-four hours an inflammation and tumefaction take place in the walls, or circumference of the wound, which close it up so completely, that a small probe cannot be introduced without force, and without giving a pain to the patient. I mention this latter, because theoretical writers on the subject talk of introducing their bullet forceps, and extracting a ball with the same facility as they would extract a ball from a large auger hole.

I saw at the period in question a pair of bullet forceps, from an instrument maker, for the army, who, no doubt, acted up to this idea, nearly as large and long as a pair of small fire tongs, and which no experienced surgeon would ever think of employing on the living body. In a vast majority of cases, no instrument larger than the common dressing forceps could be used in extracting a ball: they answered every purpose to which instruments of this kind could be applied. They are long enough, and if any objection to them can be found, on trial, it will be invariably as to their bulk, which might, with great advantage, be diminished for this purpose. When I first began to dress gun-shot wounds, and extract balls, I laid hold of the common bullet forceps, and attempted to introduce them into the wound, in which I seldom succeeded, for two reasons; the first was, because it was impossible to do so, without dilating the wound with a scalpel; and the second, because few patients will allow the excessive aggravation of pain which their unwieldly size occasions. After unavailing efforts with them, till my own patience, and that of the patient, was exhausted, I commonly finished the business with the dressing forceps.

If any person will take the trouble to compare a pair of bullet forceps, of that period, with the bullets used in our service, he will find that the diameter of the extremity of the forceps is nearly equal to the diameter of the ball; so that, admitting it practicable to insert the forceps into the wound, and to pass them along its channel till the point reaches the ball, it will then be necessary to stretch the wound to twice its diameter before the blades of the forceps can be passed over the ball, and include it in their grasp; after which the whole length of the wound is to be stretched in the same manner, in extracting the ball, and that to the very great misery of the patient. The common dressing forceps are objectionable on this account, but less so than the others, because their points are tapering and thin, and, therefore, occupy less room; and as regards firmness of grasp, it is sufficient to extract a ball out of any soft part in the human frame.

To return to the progressive condition of wounds: the tumefaction and inflammation, of which I have before spoken, generally continue, with little variation, till the fourth or fifth day; from that time a secretion of pus takes place from the walls of the wound, which continues to increase for several days, and sometimes it becomes very profuse. This secretion of pus cleanses the wound, and terminates the inflammation and tumefaction, commonly about the tenth or twelfth day. It is at this period that it is practicable to extract the ball, and pieces of clothing, which may not have been attended to at the first dressing; the wound has now a gaping orifice, and the dressing forceps may be introduced. If it be delayed much after this time, granulations rise up and close the orifice. We can then only trust to nature for expelling those foreign bodies, and it is seldom that she does not, sooner or later, bring them to the surface.

Major General Brown presented an instance of the injurious effects of portions of clothing remaining in wounds. At Bridgewater a musket ball entered somewhat in front of the trochanter major and passed out over the inguinal glands. He suffered for three weeks from inflammation and suppuration; became better, and leaving the southern shore of Lake Erie, was accommodated in an armed schooner off the Fort. He then relapsed, with great irritation and discharge of matter, which continued until a piece of woollen pantaloons carried in by the ball, was discharged. The wound then soon healed, he resumed the immediate command of the troops, and consummated his military reputation by directing the sortie of Sept. 19th.

Not an unusual point of discussion, is the comparative size of the place of entrance and of departure of a ball. It will be impossible to find a rule on this subject because the proposition is variable. If a ball pass through a part with a velocity not materially impaired, the hole for its exit will be the larger from its imperfect support in being made, but it will be found generally that the point of exit is a simple laceration, the edges of which come almost or exactly together. Cases of this kind are met with when the ball is so far expended in force that it does not penetrate the clothing a second time, but is caught by it and drops down into the boot or shoe.

The sloughing of wounds is described by many writers in such a manner as to produce the belief that the parts injured by the ball are separated from the living parts, and come out, in pretty much the same way as the lining would from the sleeve of a coat. I never saw this, or any thing like it; if a sloughing does take place, it is insensible, and so dissolved in pus, that nothing of it can be seen. Occasionally parts of the fascia and of the cellular substance are detached, but there is nothing like muscular flesh.

I should myself be more disposed to describe the process as a deliquescence of the contused parts, than a regular sloughing.

From the tenth or twelfth day, the secretion of pus gradually diminishes, granulations arise quickly, and the cicatrix commences from the circumference of the wound, contracts and advances to the centre; when it has nearly closed up, the further progress of the cicatrix is frequently checked, and it remains stationary for some time, in consequence of a small button-like fungus, which shoots up considerably above the surface of the wound. If this fungus is touched once or twice with lunar caustic, it disappears, and the wound is completely healed, generally between the 20th and 30th day. Gun-shot wounds seldom bleed much at first, or indeed at any period of their course.

I often had my fears excited by the accounts of secondary hemorrhage, proceeding from the sloughing process involving the large trunks of arteries. It is true, this event does take place sometimes, but I think it one of comparatively rare occurrence.

The discharge from a gun-shot wound is, for several days, of the most fetid and intolerable kind, and while the deadened flesh is coming away, this discharge is of a black color, mixed with yellow; it soils, very much, the dressings, and they cannot be washed clean without a great amount of labor. Owing to this we could scarcely keep a bandage in use for more than one dressing. But the expense of this profusion was very serious, from the scarcity of woven fabrics and the high price of cotton which had to be transported inland on wagons from South to North, in consequence of the strict blockade of the sea-coast. The existence of war precluded the introduction of supplies from abroad, through our sea-ports.

(To be continued.)

